# The effect of aminolaevulinic acid-induced, protoporphyrin IX-mediated photodynamic therapy on the cremaster muscle microcirculation in vivo.

**DOI:** 10.1038/bjc.1995.473

**Published:** 1995-11

**Authors:** J. Leveckis, N. J. Brown, M. W. Reed

**Affiliations:** Department of Surgical and Anaesthetic Sciences, University of Sheffield, Royal Hallamshire Hospital, UK.

## Abstract

The effect of photodynamic therapy on normal striated muscle was investigated using 30 adult male rats. Animals were divided into six groups. Three control groups received phosphate-buffered saline by gavage and violet light at 105, 178 and 300 mW cm-2 respectively. Three experimental groups received aminolaevulinic acid (ALA; 200 mg kg-1) and violet light at 105, 178 and 300 mW cm-2 respectively. After exposure of the cremaster muscle animals were allowed to equilibrate and vessel diameters and bloodflow assessed. Following photoactivation measurements were taken every 10 min over a 2 h period. Photoactivation of experimental groups at the two higher power densities resulted in an initial decrease in both arteriolar and venular diameters, and a concomitant decrease in blood flow. The magnitude of these changes and the degree of recovery by the end of the observation period was related to power density. No effects were observed in the control groups. These results suggest that microcirculatory damage may contribute to the mechanism of action of photodynamic therapy with ALA.


British Journal of Cancer (1995) 72, 1113-1119

© 1995 Stockton Press All rights reserved 0007-0920/95 $12.00

### The effect of aminolaevulinic acid-induced, protoporphyrin IX-mediated photodynamic therapy on the cremaster muscle microcirculation *in vivo*

J Leveckis, NJ Brown, MWR Reed

*Department of Surgical and Anaesthetic Sciences, University of Sheffield, Royal Hallamshire Hospital, Glossop Road, Sheffield, S10 2JF, UK.*

**Summary** The effect of photodynamic therapy on normal striated muscle was investigated using 30 adult male rats. Animals were divided into six groups. Three control groups received phosphate-buffered saline by gavage and violet light at 105, 178 and 300 mW cm$^{-2}$ respectively. Three experimental groups received aminolaevulinic acid (ALA; 200 mg kg$^{-1}$) and violet light at 105, 178 and 300 mW cm$^{-2}$ respectively. After exposure of the cremaster muscle animals were allowed to equilibrate and vessel diameters and bloodflow assessed. Following photoactivation measurements were taken every 10 min over a 2 h period. Photoactivation of experimental groups at the two higher power densities resulted in an initial decrease in both arteriolar and venular diameters, and a concomitant decrease in blood flow. The magnitude of these changes and the degree of recovery by the end of the observation period was related to power density. No effects were observed in the control groups. These results suggest that microcirculatory damage may contribute to the mechanism of action of photodynamic therapy with ALA.

**Keywords:** aminolaevulinic acid; photodynamic therapy; microcirculation; protoporphyrin IX

Photodynamic therapy (PDT) is an experimental treatment for cancer in which cell death occurs as a result of the interaction of light with a photosensitising drug. PDT using the photosensitiser haematoporphyrin derivative (HPD) has been shown to be effective in the treatment of cancers in various sites including skin (Dougherty, 1987), bronchus (Balchum, 1984) and bladder (Nseyo, 1992), often after more conventional treatments have failed. The cytoxicity of HPD-mediated PDT has been attributed to the production of reactive oxygen species, although the precise mechanism of damage is not clear. Injury to cell membranes and subcellular structures has been demonstrated *in vitro* and *in vivo* but this damage appears to be sublethal and does not fully explain the tissue destruction observed following PDT (Henderson *et al.*, 1985). A significant role for the microcirculation as a target for PDT has been proposed and disruption of blood flow in normal tissues and tumours after HPD-mediated PDT described (Star *et al.*, 1986; Wieman *et al.*, 1988).

Aminolaevulinic acid (ALA) is a naturally occurring precursor of the photosensitising agent protoporphyrin IX (PpIX) (Elder, 1983). Owing to the relatively slow conversion of PpIX into haem (Pottier *et al.*, 1986), the addition of large quantities of exogenous ALA results in the accumulation of photosensitising amounts of PpIX in many tissues (Pottier *et al.*, 1986; Kennedy *et al.*, 1990; Loh *et al.*, 1993; Leveckis *et al.*, 1994). A photodynamic effect may be achieved by the exposure of tissues to light, which correlates with the intensity of PpIX fluorescence on microscopy (Divaris *et al.*, 1990). Preliminary clinical studies have shown ALA to be an effective photosensitising agent (Kennedy *et al.*, 1990; Wolf *et al.*, 1993). It has significant potential advantages over HPD due to its low toxicity, short period of skin sensitisation and absorption after topical application (Berlin *et al.*, 1956*a*,*b*; Kennedy *et al.*, 1990). In contrast to the current photosensitisers in clinical and experimental use, ALA-induced PpIX appears to localise in cells, rather than the stroma or blood vessels of both normal and tumour tissue (Bedwell *et al.*, 1992). On the basis of these findings it has been suggested by some authors that ALA-induced, PpIX-mediated PDT may act predominantly by a different mechanism to that of HPD, with direct cytotoxicity being of more significance than microcirculatory damage (Loh *et al.*, 1992).

Correspondence: MWR Reed
Received 1 November 1994; revised 30 May 1995; accepted 7 June 1995

The aim of this study is to determine whether ALA-induced, PpIX-mediated PDT has an effect on the microcirculation. The model used was the rat cremaster preparation (Baez, 1973; Meininger *et al.*, 1987), a thin sheet of somatic muscle in which the microvasculature can be studied by *in vivo* microscopy. The rat cremaster preparation has been used extensively in the field of microcirculatory research, including study of the effect of PDT on the microcirculation (Reed *et al.*, 1988).

### Materials and methods

### *Animals and ALA administration*

Experiments were performed on male adult Wistar rats ($n = 30$) weighing 100 g, obtained from the University of Sheffield Field Laboratories. ALA for oral administration was prepared by dissolving in phosphate-buffered saline (PBS) (Sigma, Poole, Dorset, UK) resulting in a solution of pH 2.8. Three treatment groups of five animals (groups I, II and V) received ALA (200 mg kg$^{-1}$) in a volume of up to 1 ml PBS by gavage using 3 mm diameter soft silicone tubing. Precise dosing based on animal weight ($\pm$ 10 g) was achieved by varying the volume administered to each animal. Three control groups of five animals (groups III, IV and VI) received 1 ml of PBS by gavage. In all groups, dosing was carried out 4 h before photoactivation. During the period between dosing and preparation for *in vivo* microscopy, animals were returned to their cages and allowed free access to both food ('Rodent pellets', Argo Feeds, Stannington, South Yorkshire, UK) and water.

### *Preparation for* in vivo *microscopy*

One hour before photoactivation animals were anaesthetised by subcutaneous injection of a mixture of Hypnorm (Janssen Pharmaceutical) 0.5 ml kg$^{-1}$ (fentanyl citrate 0.315 mg ml$^{-1}$, fluanisone 10 mg ml$^{-1}$) and diazemuls (Dumex) 0.5 ml kg$^{-1}$ (5 mg ml$^{-1}$), 1:1 (v/v).

The left carotid artery was cannulated and connected to a pressure transducer and physiograph (Micro-Med, Louisville, USA) which monitored mean arterial blood pressure and heart rate. An oesophageal thermistor probe was inserted and connected to a digital thermometer (Fluke, Washington, USA). The animal was placed on a warming pad on a

Perspex animal board in order to maintain body temperature (35–37°C) during photoactivation and the recovery phase.

The right side of the scrotum was opened in the ventral midline and the testis and surrounding cremaster gently dissected from adjacent connective tissue. A 3/0 silk stay suture was placed in the apex of the cremaster, which was then positioned on a glass microscope slide mounted on perspex pegs attached to the animal board. The muscle was held in place by the stay suture and electrocautery used to open the cremaster along the avascular plane in the ventral midline, with care taken to avoid damage to the underlying testis. Four more stay sutures were positioned around the circumference of the cremaster to open the preparation. The dorsal connective tissue ligament between the testis and the cremaster was divided using cautery and the testis gently returned to the abdominal cavity. The cremaster muscle preparation with intact neurovascular supply was moistened with physiological saline and covered with an impermeable membrane (Saran wrap, Dow Brands, Indianapolis, IN, USA) to prevent dehydration during the period of observation.

### *Experimental protocol: photoactivation, data collection and image analysis*

The animal, warming pad and perspex board were transferred to the stage of a microscope equipped with a tungsten lamp for transmitted light microscopy and a 100 W mercury arc lamp for epi-illumination (Leitz, Germany). After transfer a further thermistor was placed under the edge of the cremaster and connected to the second channel of the thermometer. Animals were allowed an equilibration period of 30 min before photoactivation. During this time the preparation was briefly scanned with low-level transmitted light and a suitable 'area of interest' (AOI) containing second order arterioles and venules selected for study.

During each period of observation of the AOI, images of the preparation were obtained via a $\times$ 10 lens, using a charged-coupled device (CCD) camera (model KP-161, Hitachi, Japan), displayed on a high-resolution monitor (Sony PVM-1443) and recorded on video (Sony SLV-373-UB) for later off-line analysis. At the end of the equilibration period the AOI was observed for 30 s to obtain baseline values for vessel diameters. A bandpass filter interposed into the light path of the mercury arc lamp permitted violet (390–460 nm) light to be selected for epi-illumination and photoactivation, using the major absorbance peak of PpIX (405 nm). The power output was determined immediately before activation and at the end of each experiment using an optometer. Treatment times were altered as necessary after measuring the power output, to achieve a constant energy density of 100 J cm$^{-2}$ in all groups. The power output of the system as described was 16 mW: in order to achieve the lower power densities required, 5% and 30% neutral density filters were interposed between the light source and objective lens.

All groups received violet light to the AOI at a constant energy density of 100 J cm$^{-2}$ but at three different rates of delivery (power densities). Animals in groups I and III received 105 mW cm$^{-2}$, groups II and IV 178 mW cm$^{-2}$, and groups V and VI 300 mW cm$^{-2}$. During photoactivation images were recorded continuously using low-level transmitted light. After photoactivation further recordings of the area of interest were taken for 30 s at 10 min intervals for 2 h. Qualitative changes in blood flow were noted and quantitative changes in vessel diameters measured using computerised image analysis software (Image Pro Plus, Media Cybernetics, USA) preloaded on an IBM-compatible PC (Vig IV 25, Viglen, London, UK), calibrated to produce direct measurements in micrometres. The parameter measured to record vessel diameter was the red blood cell column diameter (RBCCD). Recordings of RBCCD were made every minute during photoactivation and every 10 min thereafter. The ALA and light dose groups are summarised in Table I.

### *Statistical analysis*

Since vessel diameters between animals varied, results are expressed in terms of percentage change in diameter compared to the prephotoactivation diameter (by definition 100%). Differences in vessel diameter between control and treatment groups and between prephotoactivation vessel diameter and diameter during photoactivation and the recovery phase were compared using the Mann–Whitney *U*-test for non-parametric data. Differences were considered significant at $P<0.05$.

### Results

### *Arteriolar response to ALA-induced, PpIX-mediated PDT*

No significant change in vessel diameter was observed in the ALA-treated animals during or after photoactivation with violet light at 105 mW cm$^{-2}$ (Figure 1a).

At 178 mW cm$^{-2}$ a rapid reduction in vessel diameter was observed in the ALA-treated group, which reached significance 4 min into the period of photoactivation ($P<0.02$). The maximum decrease in vessel diameter (to 22.8% of pretreatment diameter) occurred by the end of photoactivation. During the recovery phase there was a gradual increase in vessel diameter in the ALA-treated group, which at 2 h was not significantly different from the pretreatment value (Figure 1b).

At 300 mW cm$^{-2}$ a rapid decrease in vessel diameter was observed in the ALA-treated group, which reached significance 2 min into the period of photoactivation ($P<0.02$). The maximum decrease in vessel diameter (to 18% of pretreatment diameter) occurred by the end of photoactivation. During the recovery phase there was a gradual increase in vessel diameter in the ALA-treated group, which by 70 min was not significantly different from the pretreatment value (Figure 1c).

No significant change in vessel diameters was observed in the three control groups receiving light but no ALA.

### *Venular response to ALA-induced, PpIX-mediated PDT*

There was no significant change in vessel diameter in the ALA-treated group during photoactivation with violet light at 105 mW cm$^{-2}$. A small but significant reduction in vessel diameter (to 91.8% of pretreatment diameter), lasting 40 min

**Table I** Treatment groups: ALA dose, light dose, and photoactivation time

| | Groups *I* | *II* | *III* | *IV* | *V* | *VI* |
|---|---|---|---|---|---|---|
| ALA (mg kg$^{-1}$) | 200 | 200 | – | – | 200 | – |
| Fluorescent light (mW cm$^{-2}$) | 105 | 178 | 105 | 178 | 300 | 300 |
| Transmitted light | + | + | + | + | + | + |
| Treatment time (min) | 17.4 | 9.4 | 17.4 | 9.4 | 5.5 | 5.5 |

was observed in the ALA-treated group 20 min after the end of photoactivation ($P<0.02$). At the end of the recovery period vessel diameters returned to their pretreatment values (Figure 2a).

There was no significant change in vessel diameter in the ALA-treated group during photoactivation with violet light at 178 mW cm$^{-2}$. A reduction in vessel diameter however occurred during the early part of the recovery phase in the ALA-treated group, reaching a minimum of 74% of pretreatment diameter 20 min after the end of photoactivation ($P<0.02$). The reduction was maintained for 40 min after photoactivation, following which vessel diameters returned to pretreatment values (Figure 2b).

A reduction in vessel diameter was observed in the ALA-treated group, which commenced 4 min into photoactivation with violet light at 300 mW cm$^{-2}$. This reached a maximum

**Figure 1** Arteriolar response to ALA-induced, PpIX-mediated PDT. Violet light at 105 mW cm$^{-2}$ (**a**) 178 mW cm$^{-2}$ (**b**) 300 mW cm$^{-2}$ (**c**) $T_0$ = end of period of photoactivation. O, controls; ▼, treated animals receiving ALA 200 mg kg$^{-2}$ 4 h before photoactivation; mean ± s.e.m., $n = 5$ for each value. Horizontal bar indicates period of photoactivation.

of 53% of the pretreatment value 40 min after the end of photoactivation and was maintained throughout most of the recovery period, only returning to the pretreatment value at 110 min (Figure 2c).

No significant change in vessel diameter was observed in the three control groups either during photoactivation or the recovery period.

*Capillary and flow responses to ALA-induced, PpIX-mediated PDT*

At 105 mW cm$^{-2}$ all preparations in the ALA-treated group exhibited mild irritability during photoactivation with slowing of capillary flow. During the recovery period normal capillary flow returned in four of the five cremasters. In the

**Figure 2** Venular response to ALA-induced, PpIX-mediated PDT. Violet light at 105 mW cm$^{-2}$ (**a**) 178 mW cm$^{-2}$ (**b**) 300 mW cm$^{-2}$ (**c**) $T_0$ = end of period of photoactivation. O, controls; ▼, treated animals receiving ALA 200 mg kg$^{-2}$ 4 h before photoactivation; mean ± s.e.m., $n = 5$ for each value. Horizontal bar indicates period of photoactivation.

fifth preparation a reduction in arterial flow was observed during treatment which was maintained to the end of the recovery phase.

At 178 mW $cm^{-2}$ a rapid reduction in capillary flow was observed in all preparations in the ALA-treated group. At the end of the recovery period complete cessation of capillary flow was still evident in three of the five preparations, with very sluggish return of flow in two. Arterial blood flow reduced progressively during photoactivation, with partial recovery only in four cases.

At 300 mW $cm^{-2}$ a rapid reduction in capillary flow was observed in all preparations in the ALA-treated group, with complete cessation of flow by the end of photoactivation. At the end of the recovery period there was return of capillary flow in two of the five preparations. A reduction in arterial blood flow during photoactivation, which returned to normal at the end of the recovery period was observed in four of the five preparations. Venous blood flow was reduced during photoactivation but returned to normal in all preparations by the end of the recovery period.

No changes in blood flow were observed in the control group.

*Physiological parameters*

There were no significant differences between oesophageal temperature, cremasteric temperature, heart rate and blood pressure between the control and ALA-treated groups.

### Discussion

Direct cytotoxicity has been shown to be insufficient to explain the effects of PDT. Cells from murine tumours remaining *in situ* after PDT undergo necrosis, whereas those explanted immediately after photoactivation remain viable *in vitro*, suggesting that local tissue factors may play an important role (Henderson *et al*., 1985).

Blood flow changes resulting from PDT were first quantified by Selman *et al*. (1984) in transplantable bladder tumours treated with HPD (10 $\mu g\ g^{-1}$) and red light (630 J $cm^{-2}$). Using a radioactive microsphere technique they demonstrated a significant reduction in blood flow to tumours 10 min and 24 h after the end of photoactivation. Using an identical model, blood flow reduction was shown to be related to both light and photosensitiser dose (Selman *et al*., 1985*a*). The hypothesis that abnormal tumour vasculature might be responsible for the selectivity of PDT was challenged when Selman's group also reported similar blood flow changes in non-neoplastic tissue (Selman *et al*., 1985*b*).

Though the radioactive microsphere technique and laser doppler velocimetry (Wieman *et al*., 1988) clearly demonstrated the quantitative reduction in blood flow in normal and neoplastic tissue after PDT, direct observation of the microcirculation is required to determine the pathogenesis of the vascular changes. Preliminary observations were reported by Castellani *et al*. (1963) who described sequential changes to the microcirculation in the frog tongue and rabbit mesentery after treatment with haematoporphyrin and light.

The first detailed *in vivo* observations of the microcirculatory changes after HPD phototherapy were made using sandwich observation chambers (Star *et al*., 1986). 'Blanching' of the capillary bed in a rat mammary tumour was observed during photoactivation, followed by a reduction in the red blood cell column diameter (RBCCD) in larger vessels. Capillary perfusion eventually returned unless high light doses ($>$70 J $cm^{-2}$) were used. This resulted in obvious tumour necrosis 1–2 days after phototherapy. Significantly, tumour regrowth occurred unless the circulation in a margin of normal tissue adjacent to the tumour was also destroyed. Normal tissue vessels were more resistant but eventually underwent similar changes when higher light doses were used, with apparent vasoconstriction (reduction in RBCCD), platelet thrombosis, oscillatory and even reversed flow being observed.

The rat cremaster preparation has been used to study the mechanism of action of PDT, in particular the sequence of microcirculatory changes occurring during and shortly after photoactivation. Activation by either blue or green light 30 min after the intra-arterial injection of dihaematoporphyrin ether (DHE) results in a rapid reduction in both arteriolar and venular diameter, an effect which is both light and photosensitiser dose dependent. In conjunction with the formation and embolisation of platelet thrombi this leads to stasis in 80% of arterioles and 90% of venules, with reperfusion occurring in only 20% of arterioles at 2 h (Reed *et al*., 1988). Similar changes have been demonstrated in implanted tumour vessels after DHE-mediated PDT, with vasoconstriction predominating in arterioles and thrombosis in venules (Reed *et al*., 1989*a*). Sequential changes observed with the light or electron microscope include early margination of neutrophils, platelet aggregation, mitochondrial degeneration, damage to endothelial cells and both vascular and skeletal myocytes (Chaudhuri *et al*., 1987; Tseng *et al*., 1988, Reed *et al*., 1989*a*). A progressive increase in venular permeability to albumin occurs, resulting in interstitial oedema (Fingar *et al*., 1992). The resultant reduction in blood flow, in particular that demonstrated in the periphery of tumours, and the consequent hypoxia is thought to be of great significance in the aetiology of the necrosis seen after PDT (Reed *et al*., 1989*b*).

These cellular changes do not however explain the very rapid initial microcirculatory responses observed following light administration. Recent studies using the rat cremaster suggest that vasoactive agents such as prostaglandins or thromboxane may mediate the early response, since administration of cyclooxygenase inhibitors such as indomethacin, acetyl salicylic acid and the specific thromboxane $A_2$ receptor antagonist SQ29548 prevent the vascular stasis (Reed *et al*., 1989*c*, 1991; Fingar *et al*., 1990).

It is clear from the evidence presented above that vascular damage plays a significant role in PDT. But this phenomenon is not unique to the 'classical' photosensitisers HPD and DHE. Sensitive fluorescence microscopy of frozen sections taken after sensitisation with phthalocyanine has demonstrated localisation of fluorescence to well-vascularised areas of stroma in the rat colon (Barr *et al*., 1988), rat bladder (Pope *et al*., 1991*a*), and hamster pancreas (Chatlani *et al*., 1992). Using a radioactive microsphere technique, blood flow to transplantable bladder tumours has been shown to be markedly reduced after treatment with chloroaluminium tetrasulphophthalocyanine and light (Selman *et al*., 1986), indicating that HPD and phthalocyanines have similar effects on the microvasculature.

In contrast, studies of the distribution of PpIX fluorescence suggest that ALA-induced, PpIX-mediated phototoxicity may have a fundamentally different mode of action, with direct cytotoxicity predominating over vascular effects. The evidence for this hypothesis is based on studies of ALA-induced, PpIX fluorescence detected by sensitive fluorescence microscopy of thin frozen sections. PpIX fluorescence in hollow organs including colon, bladder and stomach has been detected principally within cells, in contrast to the perivascular distribution observed with phthalocyanines (Loh *et al*., 1992, 1993; Bedwell *et al*., 1992). These findings have led to the suggestion that ALA passes from the circulation directly through the endothelial cells of capillaries, into the extracellular fluid and diffuses into target cells, where it is then converted to PpIX (Kennedy and Pottier, 1992). The advantages suggested for this mode of action include:

(1) reduced likelihood of disruption to supporting tissue and vascular stroma;
(2) improved healing with less scarring;
(3) selective eradication of small nests of tumour cells without damage to adjacent normal tissue, provided differential photosensitisation exists between the two (Loh *et al*., 1993).

The results presented here clearly conflict with the hypothesis derived from *in vitro* fluorescence distribution studies. ALA-induced, PpIX-mediated PDT has been shown to have a profound effect on the microcirculation in these *in vivo* studies. At the two higher power densities there was a rapid power density-dependent reduction in arteriolar RBCCD which reached a maximum at the end of the period of photoactivation. Venular RBCCD was also affected but the changes were quantitatively and qualitatively different. Maximum reduction in venular diameter was less than that demonstrated in equivalent order arterioles and the maximum effect occurred after the end of the period of photoactivation. Changes in medium order vessel diameters were accompanied by a reduction in flow in arterioles, venules and capillaries. Complete or near complete stasis of capillary flow was observed even at the end of the 2 h recovery period in 60% of the preparations receiving the two higher light doses, suggesting that in many cases these microcirculatory changes are irreversible.

The quantitative and qualitative changes in arteriolar and venular RBCCD identified here are similar to those described previously in this model using DHE as the photosensitiser. The reported maximum reduction in arteriolar diameter in the cremaster after activation of DHE with green light (200 mW $cm^{-2}$, 120 J $cm^{-2}$) is 20–40% of pretreatment diameter. This occurs within the first 30 s of photoactivation, reaching a maximum at 3 min. The reduction in venular RBCCD is delayed compared with the arteriolar response and reaches a maximum of 60% of the pretreatment value (Reed *et al*., 1988). Using pooled data from the two higher power density groups in this series (178 and 300 mW $cm^{-2}$) the corresponding reductions with ALA were to 20% of pretreatment value for the arterioles and 60% for the venules. Reperfusion occurred by 2 h in 50% of the arterioles and 25% of the venules in Reed's study using DHE (Reed *et al*., 1988). With ALA, reperfusion similarly occurred in 50% of arterioles by 2 h. In contrast, after an initial reduction, venous flow appeared to return to normal by the end of the recovery period.

These findings strongly support recent evidence for a microcirculatory effect from *in vivo* and *ex vivo* work. Using an $^{86}$RbCl extraction method to compare blood flow in a rat fibrosarcoma model, Roberts *et al*. (1994) have demonstrated that phototherapy based on either polyhaematoporphyrin (PHP) or ALA results in a significant reduction in tumour vascular perfusion. Rapid initial reductions were observed (of 93% and 80% respectively), followed by partial recovery over days. The pattern of change in both tumour vascular perfusion and tumour growth was remarkably similar for both agents, though recovery was more rapid after ALA-induced PDT. Sandwich chamber observations using ALA-induced PDT have demonstrated the rapid onset of microcirculatory damage in both normal rat skin and subcutaneously implanted tumour tissue after photoactivation with green light at 100 J $cm^{-2}$, 100 mW $cm^{-2}$. The pattern of change in severity of vascular damage was similar for skin and tumour, reaching a maximum at the end of light activation (van der Veen *et al*., 1994). In contrast, we observed very little change at this light dose and power density. This may be due to differences in PpIX kinetics between the two tissues studied, differences in the timing of light activation (60–100 min *vs* 240 min) or increased resistance of normal vessels to ALA-induced PDT compared with tumour microvasculature. Further evidence directly conflicting with the fluorescence microscopy studies previously cited (Loh *et al*., 1992, 1993; Bedwell *et al*., 1992) and corroborating the findings described in this paper has been given by Roberts *et al*. (1994). Using sensitive fluorescence microscopy in conjunction with the vascular marker H33342, ALA-induced PpIX fluorescence has been detected not only in the cytoplasm of tumour cells but also in cells of the tumour vasculature. This latter observation being in keeping with the previously described correlation between ALA-induced PpIX fluorescence and the location of phototoxic damage (Divaris *et al*., 1990).

In contrast to previous studies, we have examined the effect of varying the rate of energy delivery (power density) rather than total energy. At least in the case of ALA-mediated PDT, the rate of light energy delivery may be as significant a variable as the total energy used. Additionally (at least in this particular model) there is a power density threshold which must be exceeded for a significant PDT effect to occur.

The mechanism of these microcirculatory changes was not studied in detail in our experiments, but a number of observations may be made. Significant macromolecular leakage is likely to be part of the response with ALA since interstitial oedema was evident in the preparations even at the lowest light dose. The question of whether the reduction in RBCCD is due primarily to vasoconstriction or to platelet thrombosis has not been addressed specifically and is the subject of further studies. However, in a number of preparations platelet thrombi were observed, in particular within venules. The detachment and subsequent distal embolisation of these thrombi occasionally resulted in reperfusion in the observed vessel. This appeared to be independent of any obvious change in vessel diameter. It does seem likely therefore that in common with phototherapy using DHE, a combination of vasoconstriction and thrombosis is responsible for the profound changes in the microcirculation during ALA-induced, PpIX-mediated PDT.

The significance of vascular changes in PDT is evident from the studies carried out by other investigators and those described above. But the microcirculation is to some extent a 'non-specific' target and damage to it may be expected to have deleterious effects in certain circumstances. This is especially significant in the case of the urinary bladder, since if photosensitiser concentration and or light dose within the detrusor are not controlled, smooth muscle necrosis may result from this microcirculatory damage. In clinical bladder PDT this has been shown to lead to fibrous contracture of the bladder and upper tract complications in some cases (Harty *et al*., 1989). Significantly an association has been described between local vascularity and the extent of cellular injury following PDT with HPD in transitional cell carcinoma (Schulock *et al*., 1984), and in addition there is now evidence that microcirculatory changes also occur in the bladder after PDT (Reed *et al*., 1989*d*). In comparison with the rat cremaster, studies of the urinary bladder microcirculation are sparse and the model is not as well developed. However, similar microcirculatory changes to those in the cremaster have been observed using electron microscopy, with pavementing of leucocytes, platelet aggregation, erythrocyte packing and endothelial cell damage. Detrusor myocyte (smooth muscle) injury also occurs and is mainly located near sites of severe vascular injury, suggesting a significant relationship between these two events (Reed *et al*., 1989*d*; Tseng *et al*., 1991).

This study has demonstrated that ALA-induced PDT has profound effects on the normal microcirculation, including a reduction in arteriolar and venular diameter, decreased blood flow and capillary shutdown, which in some cases is non-recoverable. These results suggest that microcirculatory damage may contribute to the mechanism of action of photodynamic therapy with ALA.

**Acknowledgement**
This study was carried out with the aid of a grant from the Trustees of the Former United Sheffield Hospitals.

### References

BAEZ J. (1973). An open cremaster preparation for the study of blood vessels by *in vivo* microscopy. *Microvascular Res.*, **5**, 384–395.

BALCHUM OJ, DOIRON DR AND HUTH GC. (1984). Photoradiation therapy for endobronchial lung cancers employing the photodynamic action of haematoporphyrin derivative. *Lasers Surg. Med.*, **4**, 13–30.

BARR H, TRALAU CJ, MACROBERT AJ, MORRISON I, PHILLIPS D AND BOWN SG. (1988). Fluorescence photometric techniques for determination of microscopic tissue distribution of phthalocyanine photosensitisers for photodynamic therapy. *Lasers Med. Sci.*, **3**, 81–86.

BEDWELL J, MACROBERT AJ, PHILLIPS D AND BOWN SG. (1992). Fluorescence distribution and photodynamic effect of ALA-induced PpIX in the DMH rat colonic tumour model. *Br. J. Cancer*, **65**, 818–824.

BERLIN NI, NEUBERGER A AND SCOTT JJ. (1956*a*). The metabolism of delta-Aminolaevulinic acid. 1. Normal pathways studied with the aid of $^{15}N$. *Biochemistry*, **64**, 80–90.

BERLIN NI, NEUBERGER A AND SCOTT JJ. (1956*b*). The metabolism of delta-Aminolaevulinic acid. 2. Normal pathways studied with the aid of $^{14}C$. *Biochemistry*, **64**, 90–100.

CASTELLANI A, PACE GP AND CONCIOLI M. (1963). Photodynamic effect of haematoporpyrin on blood microcirculation. *J. Pathol. Bact.*, **86**, 99–102.

CHATLANI PT, NUUTINEN PJO, TODA N, MACROBERT AJ, BEDWELL J AND BOWN SG. (1992). Selective necrosis in hamster pancreatic tumours using photodynamic therapy with phthalocyanine photosensitisation. *Br. J. Surg.*, **79**, 786–790.

CHAUDHURI K, KECK RW AND SELMAN SH. (1987). Morphological changes of tumor microvasculature following haematoporphyrin derivative sensitized photodynamic therapy. *Photochem. Photobiol.*, **46**, 823–827.

DOUGHERTY TJ. (1987). Photosensitisers: Therapy and detection of malignant tumours. *Photochem. Photobiol.*, **45**, 879–889.

DIVARIS DXG, KENNEDY JC AND POTTIER RH. (1990). Phototoxic damage to sebaceous glands and hair follicles of mice after systemic administration of 5-Aminolevulinic acid correlates with localized protoporphyrin production. *Am. J. Pathol.*, **136**, 891–897.

ELDER GH. (1983). Haem synthesis and the porphyrias. In *Scientific Foundations of Clinical Biochemistry*, Williams DL and Marks V (eds) pp. 175–186. Heinemann: London.

FINGAR VH, WIEMAN TJ AND DOAK KW. (1990). Role of thromboxane and prostacycline release on photodynamic therapy-induced tumour destruction. *Cancer Res.*, **50**, 2599–2603.

FINGAR VH, WIEMAN TJ, WIEHLE SA AND CERRITO PB. (1992). The Role of microvascular damage in photodynamic therapy: The effect of treatment on vessel constriction, permeability, and leukocyte adhesion. *Cancer Res.*, **52**, 4914–4921.

HARTY JI, AMIN M, WIEMAN TJ, TSENG MT, ACKERMAN D AND BROGHAMER W. (1989). Complications of whole bladder dihaematoporphyrin ether photodynamic therapy. *J. Urol.*, **141**, 1341–1346.

HENDERSON BW, WALDOW SM, MANG TS, POTTER WR, MALONE PB AND DOUGHERTY TJ. (1985). Tumor destruction and kinetics of tumor cell death in two experimental mouse tumors following photodynamic therapy. *Cancer Res.*, **45**, 572–576.

KENNEDY JC AND POTTIER RH. (1992). Endogenous protoporphyrin IX, a clinically useful photosensitiser for photodynamic therapy. *J. Photochem. Photobiol. B: Biol.*, **14**, 275–292.

KENNEDY JC, POTTIER RH AND PROSS DC. (1990). Photodynamic therapy with endogenous protoporphyrin IX: Basic principles and present clinical experience. *J. Photochem. Photobiol. B: Biol.*, **6**, 143–148.

LEVECKIS J, BURN JL, BROWN NJ AND REED MWR. (1994). Kinetics of endogenous protoporphyrin IX induction by aminolaevulinic acid: Preliminary studies in the bladder. *J. Urol.*, **152**, 550–553.

LOH CS, BEDWELL J, MACROBERT AJ, KRASNER N, PHILLIPS D AND BOWN SG. (1992). Photodynamic therapy of the normal rat stomach: A comparative study between di-sulphonated aluminium phthalocyanine and 5-aminolaevulinic acid. *Br. J. Cancer*, **66**, 452–462.

LOH CS, MACROBERT AJ, BEDWELL J, REGULA J AND BOWN SG. (1993). Oral versus intravenous administration of 5-aminolaevulinic acid for photodynamic therapy. *Br. J. Cancer*, **68**, 41–51.

MEININGER GA, FEHR KL AND YATES MB. (1987). Anatomic and haemodynamic characteristics of the blood vessels feeding the cremaster muscle of the rat. *Microvasc. Res.*, **33**, 81–97.

NSEYO UO. (1992). Photodynamic therapy. *Urol. Clin. North Am.* **19**, 591–599.

POPE AJ, MACROBERT D AND BOWN SG. (1991*a*). The detection of phthalocyanine fluorescence in normal rat bladder wall using sensitive digital imaging microscopy. *Br. J. Cancer*, **64**, 875–879.

POTTIER RH, CHOW YFA, LaPLANTE J-P, TRUSCOTT TG, KENNEDY JC AND BEINER LA. (1986). Non-invasive technique for obtaining fluorescence excitation and emission spectra *in vivo*. *Photochem. Photobiol.*, **44**, 679–687.

REED MWR, MILLER FN, WIEMAN TJ, TSENG MT AND PIETSCH CG. (1988). The effect of photodynamic therapy on the microcirculation. *J. Surg. Res.*, **45**, 452–459.

REED MWR, WIEMAN TJ, SHUSCHKE DA, TSENG MT AND MILLER FN. (1989*a*). A comparison of the effects of photodynamic therapy on normal and tumour blood vessels in the rat microcirculation. *Radiat. Res.*, **119**, 542–552.

REED MWR, MULLINS AP, ANDERSON GL, MILLER F AND WIEMAN TJ. (1989*b*). The effect of photodynamic therapy on tumour oxygenation. *Surgery*, **106**, 94–99.

REED MWR, WIEMAN TJ, DOAK KW, PIETSCH CG AND SHUSCHKE DA. (1989*c*). The microvascular effects of photodynamic therapy: Evidence for a possible role of cyclooxegenase inhibitors. *Photochem. Photobiol.*, **50**, 419–423.

REED MWR, SCHUSCHKE DA, ACKERMANN DM, HARTY JI, WIEMAN TJ AND MILLER FN. (1989*d*). The response of the rat urinary bladder microcirculation to photodynamic therapy. *J. Urol.*, **142**, 865–868.

REED MWR, SCHUSCHKE DA AND MILLER FN. (1991). Prostanoid antagonists inhibit the response of the microcirculation to 'early' photodynamic therapy. *Radiat. Res.*, **127**, 292–296.

ROBERTS DJH, CAIRNDUFF F, DRIVER I, DIXON B AND BROWN SB. (1994). *Int. J. Oncol.*, **5**, 763–768.

SCHULOCK JR, KLAUNIG JE, SELMAN SE, SCHAFER PJ AND GOLDBLATT PJ. (1984). Ultrastructural effects of combined haematoporphyrin derivative photodynamic therapy on normal and neoplastic rat bladder cells. *Am. J. Pathol.*, **122**, 277–283.

SELMAN SH, KREIMER-BIRNBAUM M, KLAUNIG JE, GOLDBLATT PJ, KECK RW AND BRITTON SL. (1984). Blood flow in transplantable bladder tumours treated with haematoporphyrin derivative and light. *Cancer Res.*, **44**, 1924–1927.

SELMAN SH, MILLIGAN AJ, KREIMER-BIRNBAUM M, KECK RW, GOLDBLATT PJ AND BRITTON SL. (1985*a*). Haematoporphyrin derivative photochemotherapy of experimental bladder tumours. *J. Urol.*, **133**, 330–333.

SELMAN SH, KREIMER-BIRNBAUM M, GOLDBLATT PJ, ANDERSON TS, KECK RW AND BRITTON SL. (1985*b*). Jejunal blood flow after exposure to light in rats injected with haematoporphyrin derivative. *Cancer Res.*, **45**, 6425–6427.

SELMAN SH, KREIMER-BIRNBAUM M, CHAUDHURI K, GARBO GM, SEAMAN DA, KECK RW, BEN-HUR E AND ROSENTHAL I. (1986). Photodynamic treatment of transplantable bladder tumours in rodents after pretreatment with chloroaluminium tetrasulfophthalocyanine. *J. Urol.*, **136**, 141–145.

STAR WM, MARIJNISSEN HPA, VAN DEN BERG-BLOK AE, VERSTEEG JAC, FRANKEN KAP AND REINHOLD HS. (1986). Destruction of rat mammary tumour and normal tissue microcirculation by haematoporphyrin derivative photoradiation observed *in vivo* in sandwich observation chambers. *Cancer Res.*, **46**, 2532–2540.

TSENG MT, REED MWR, ACKERMANN DM, SCHUSCHKE DA, WIEMAN TJ AND MILLER FN. (1988). Photodynamic therapy induced ultrastructural alterations in the microvasculature of the rat cremaster. *Photochem. Photobiol.*, **48**, 675–681.

TSENG MT, SHUSCHKE DA, REED MWR, HARTY JI, WIEMAN J AND FINGAR VH. (1991). The influence of photodynamic therapy on the ultrastructure of the normal rat bladder. *J. Photochem. Photobiol. B: Biol.*, **9**, 295–305.

VAN DER VEEN N, VAN LEENGOED HLLM AND STAR WM. (1994). *In vivo* fluorescence kinetics and photodynamic therapy using 5-aminolaevulinic acid-induced porphyrin: increased damage after multiple irradiations. *Br. J. Cancer*, **70**, 867–872.

WIEMANN TJ, MANG T, FINGAR VH, GILL TG, REED MWR, COREY T, NGUYEN VQ AND RENDER ER. (1988). Effect of photodynamic therapy on blood flow in normal and tumour vessels. *Surgery*, **104**, 512–517.

WOLF P, RIEGER E AND KERL H. (1993). Topical photodynamic therapy with endogenous porphyrins after application of 5-aminolevulinic acid. An alternative treatment modality for solar keratoses, superficial squamous cell carcinomas, and basal cell carcinomas. *J. Am. Acad. Dermatol.*, **28(1)**, 17–21.


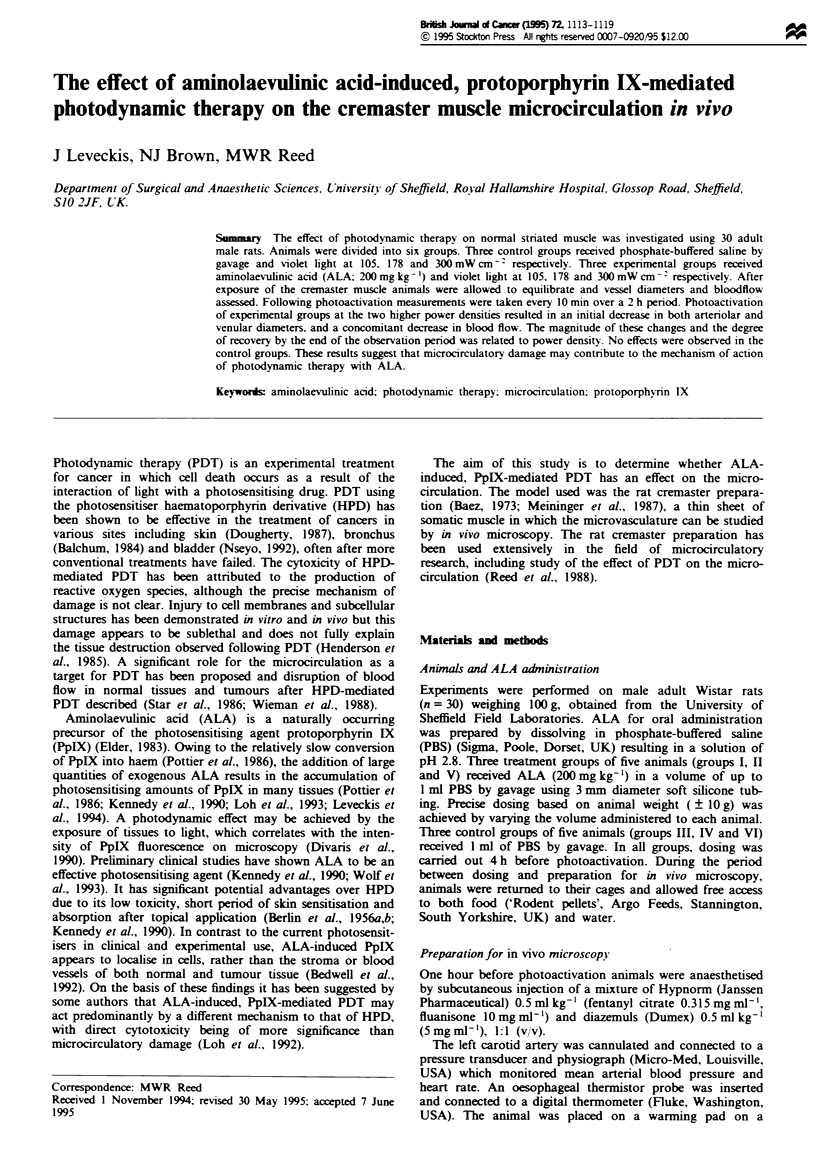

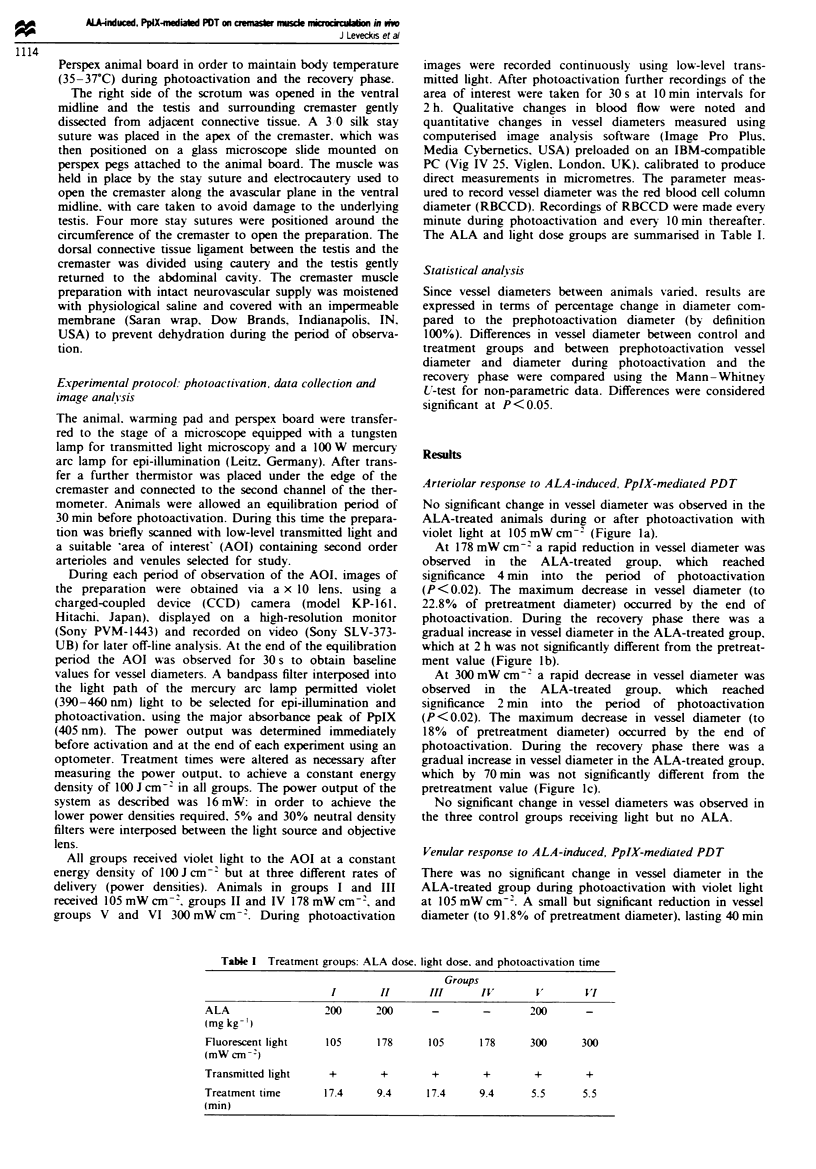

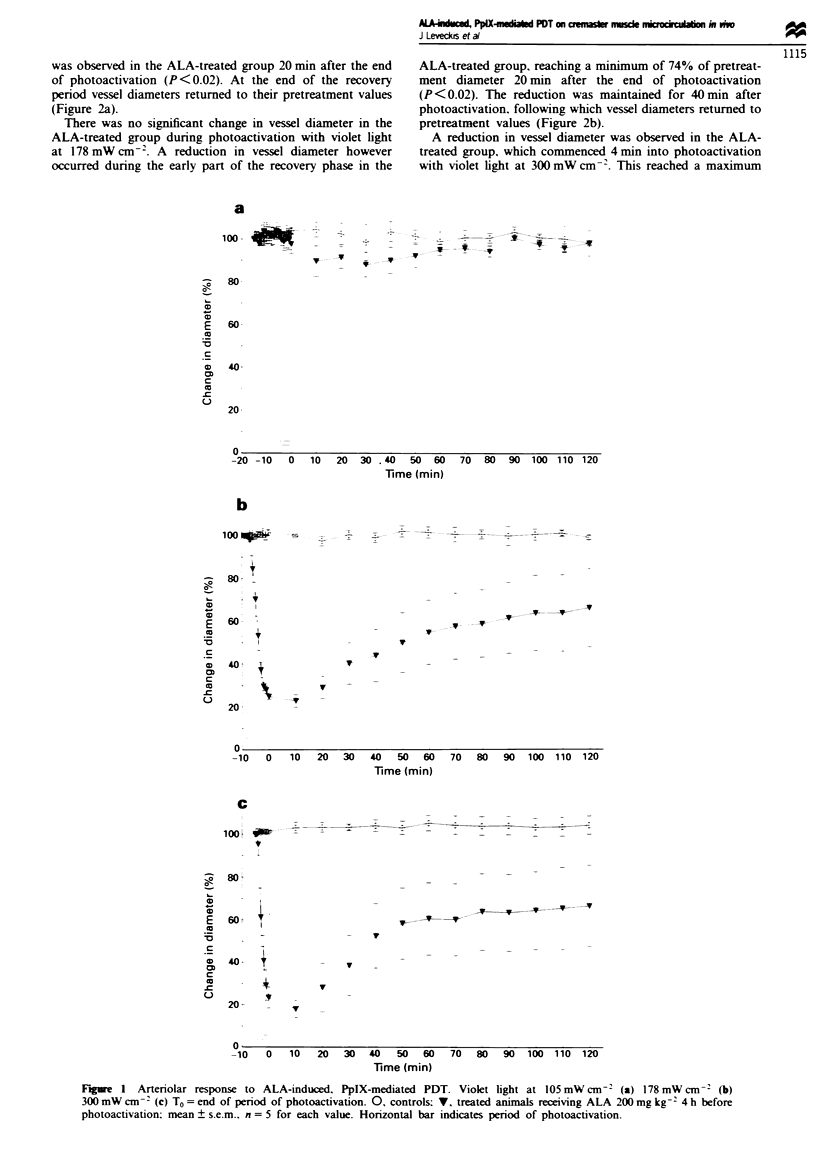

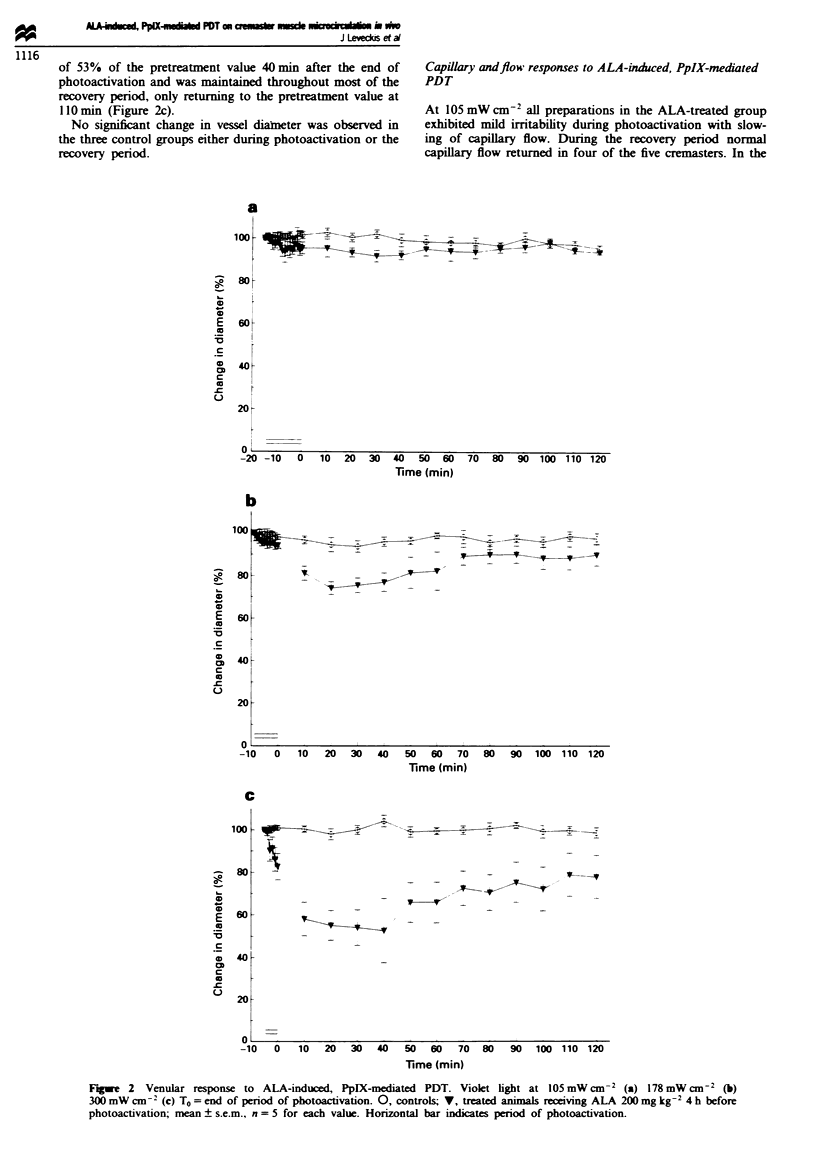

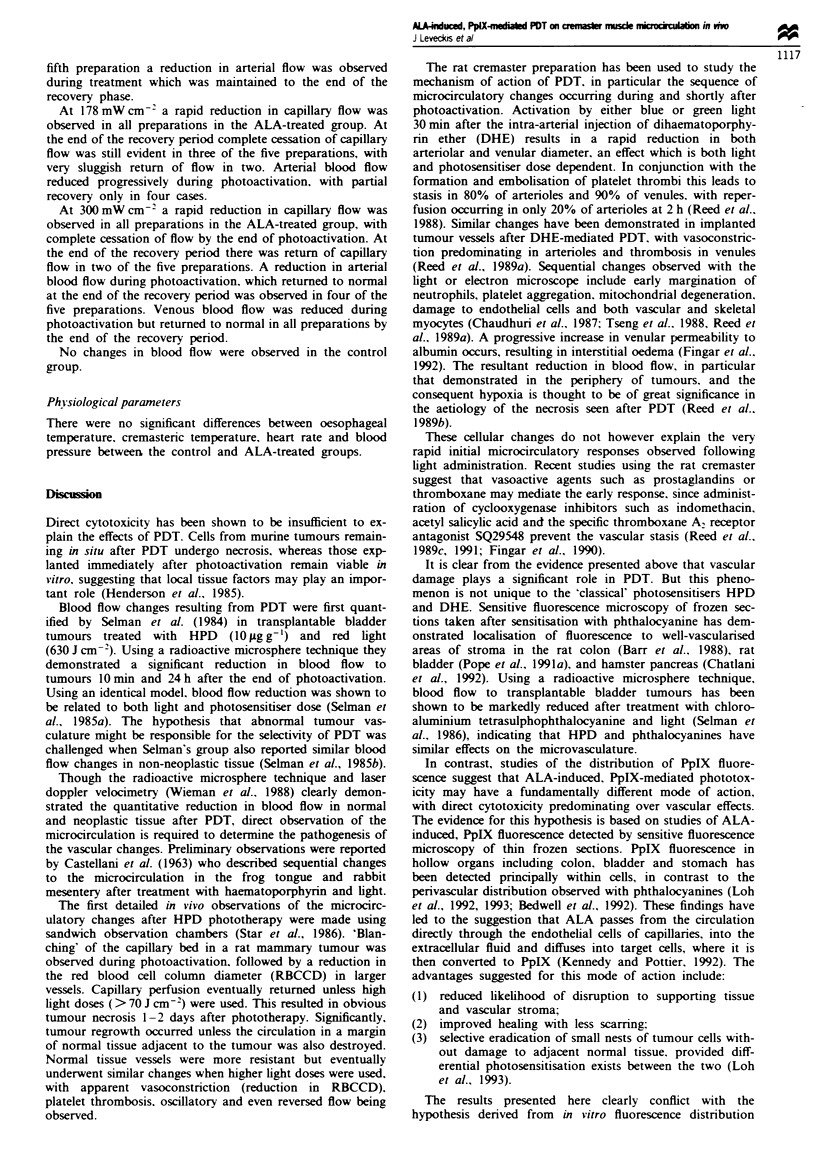

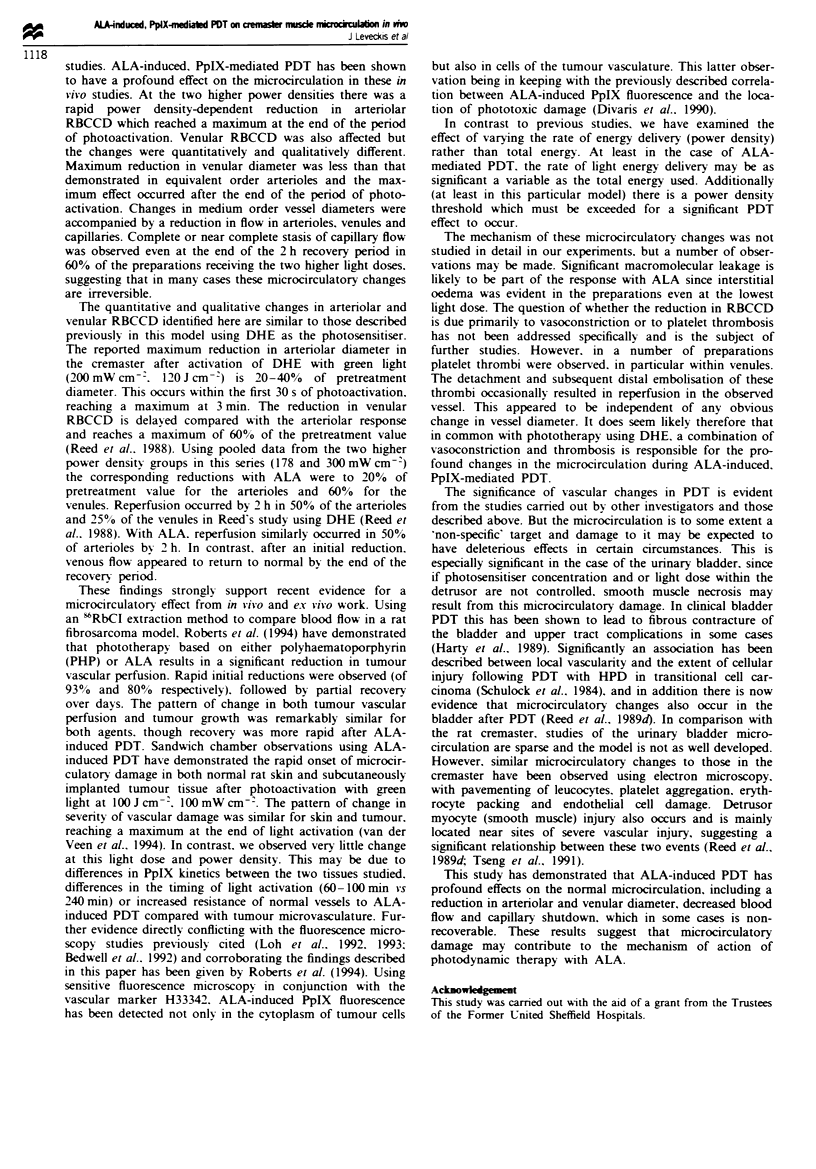

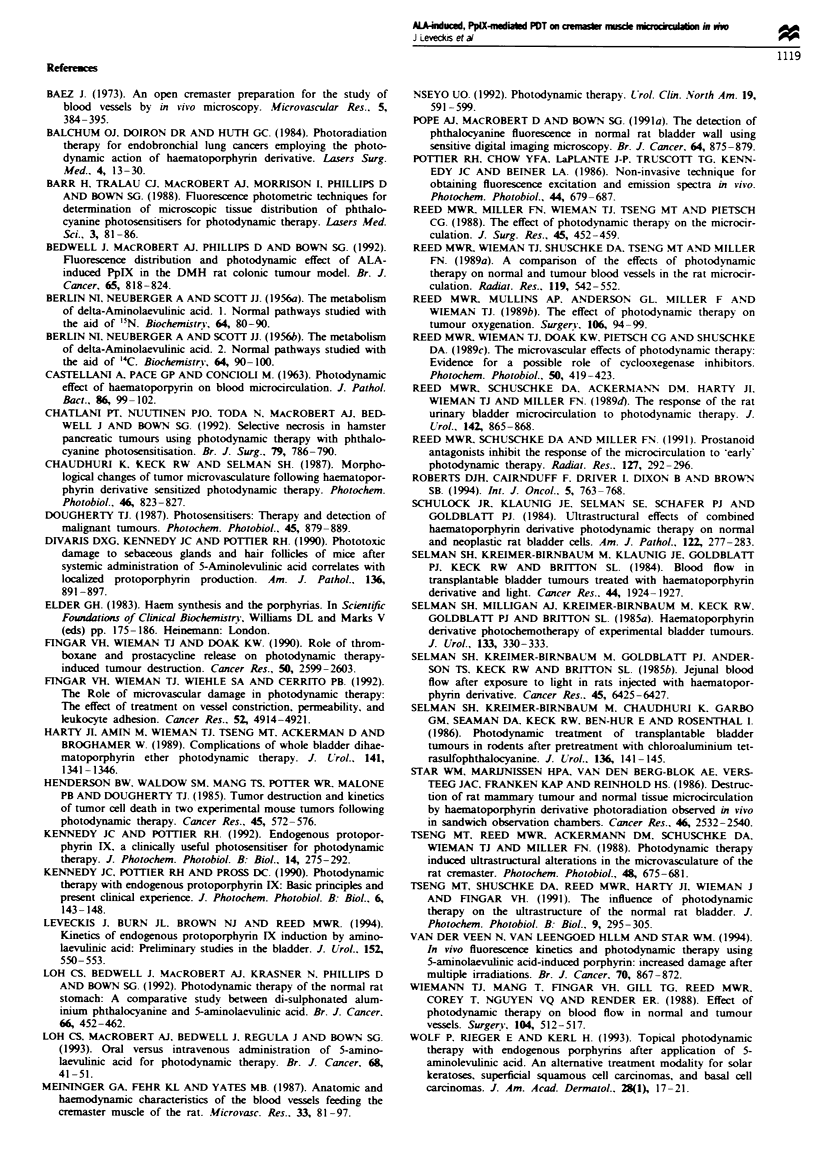


## References

[OCR_00910] BERLIN N. I., NEUBERGER A., SCOTT J. J. (1956). The metabolism of delta -aminolaevulic acid. 1. Normal pathways, studied with the aid of 15N.. Biochem J.

[OCR_00913] BERLIN N. I., NEUBERGER A., SCOTT J. J. (1956). The metabolism of delta -aminolaevulic acid. 2. Normal pathways, studied with the aid of 14C.. Biochem J.

[OCR_00886] Baez S. (1973). An open cremaster muscle preparation for the study of blood vessels by in vivo microscopy.. Microvasc Res.

[OCR_00891] Balchum O. J., Doiron D. R., Huth G. C. (1984). Photoradiation therapy of endobronchial lung cancers employing the photodynamic action of hematoporphyrin derivative.. Lasers Surg Med.

[OCR_00902] Bedwell J., MacRobert A. J., Phillips D., Bown S. G. (1992). Fluorescence distribution and photodynamic effect of ALA-induced PP IX in the DMH rat colonic tumour model.. Br J Cancer.

[OCR_00920] CASTELLANI A., PACE G. P., CONCIOLI M. (1963). Photodynamic effect of haematoporphyrin on blood microcirculation.. J Pathol Bacteriol.

[OCR_00923] Chatlani P. T., Nuutinen P. J., Toda N., Barr H., MacRobert A. J., Bedwell J., Bown S. G. (1992). Selective necrosis in hamster pancreatic tumours using photodynamic therapy with phthalocyanine photosensitization.. Br J Surg.

[OCR_00931] Chaudhuri K., Keck R. W., Selman S. H. (1987). Morphological changes of tumor microvasculature following hematoporphyrin derivative sensitized photodynamic therapy.. Photochem Photobiol.

[OCR_00941] Divaris D. X., Kennedy J. C., Pottier R. H. (1990). Phototoxic damage to sebaceous glands and hair follicles of mice after systemic administration of 5-aminolevulinic acid correlates with localized protoporphyrin IX fluorescence.. Am J Pathol.

[OCR_00935] Dougherty T. J. (1987). Photosensitizers: therapy and detection of malignant tumors.. Photochem Photobiol.

[OCR_00953] Fingar V. H., Wieman T. J., Doak K. W. (1990). Role of thromboxane and prostacyclin release on photodynamic therapy-induced tumor destruction.. Cancer Res.

[OCR_00958] Fingar V. H., Wieman T. J., Wiehle S. A., Cerrito P. B. (1992). The role of microvascular damage in photodynamic therapy: the effect of treatment on vessel constriction, permeability, and leukocyte adhesion.. Cancer Res.

[OCR_00965] Harty J. I., Amin M., Wieman T. J., Tseng M. T., Ackerman D., Broghamer W. (1989). Complications of whole bladder dihematoporphyrin ether photodynamic therapy.. J Urol.

[OCR_00971] Henderson B. W., Waldow S. M., Mang T. S., Potter W. R., Malone P. B., Dougherty T. J. (1985). Tumor destruction and kinetics of tumor cell death in two experimental mouse tumors following photodynamic therapy.. Cancer Res.

[OCR_00976] Kennedy J. C., Pottier R. H. (1992). Endogenous protoporphyrin IX, a clinically useful photosensitizer for photodynamic therapy.. J Photochem Photobiol B.

[OCR_00981] Kennedy J. C., Pottier R. H., Pross D. C. (1990). Photodynamic therapy with endogenous protoporphyrin IX: basic principles and present clinical experience.. J Photochem Photobiol B.

[OCR_00987] Leveckis J., Burn J. L., Brown N. J., Reed M. W. (1994). Kinetics of endogenous protoporphyrin IX induction by aminolevulinic acid: preliminary studies in the bladder.. J Urol.

[OCR_00993] Loh C. S., Bedwell J., MacRobert A. J., Krasner N., Phillips D., Bown S. G. (1992). Photodynamic therapy of the normal rat stomach: a comparative study between di-sulphonated aluminium phthalocyanine and 5-aminolaevulinic acid.. Br J Cancer.

[OCR_01000] Loh C. S., MacRobert A. J., Bedwell J., Regula J., Krasner N., Bown S. G. (1993). Oral versus intravenous administration of 5-aminolaevulinic acid for photodynamic therapy.. Br J Cancer.

[OCR_01006] Meininger G. A., Fehr K. L., Yates M. B. (1987). Anatomic and hemodynamic characteristics of the blood vessels feeding the cremaster skeletal muscle in the rat.. Microvasc Res.

[OCR_01009] Nseyo U. O. (1992). Photodynamic therapy.. Urol Clin North Am.

[OCR_01015] Pope A. J., MacRobert A. J., Phillips D., Bown S. G. (1991). The detection of phthalocyanine fluorescence in normal rat bladder wall using sensitive digital imaging microscopy.. Br J Cancer.

[OCR_01020] Pottier R. H., Chow Y. F., LaPlante J. P., Truscott T. G., Kennedy J. C., Beiner L. A. (1986). Non-invasive technique for obtaining fluorescence excitation and emission spectra in vivo.. Photochem Photobiol.

[OCR_01023] Reed M. W., Miller F. N., Wieman T. J., Tseng M. T., Pietsch C. G. (1988). The effect of photodynamic therapy on the microcirculation.. J Surg Res.

[OCR_01048] Reed M. W., Schuschke D. A., Ackermann D. M., Harty J. I., Wieman T. J., Miller F. N. (1989). The response of the rat urinary bladder microcirculation to photodynamic therapy.. J Urol.

[OCR_01051] Reed M. W., Schuschke D. A., Miller F. N. (1991). Prostanoid antagonists inhibit the response of the microcirculation to "early" photodynamic therapy.. Radiat Res.

[OCR_01041] Reed M. W., Wieman T. J., Doak K. W., Pietsch C. G., Schuschke D. A. (1989). The microvascular effects of photodynamic therapy: evidence for a possible role of cyclooxygenase products.. Photochem Photobiol.

[OCR_01031] Reed M. W., Wieman T. J., Schuschke D. A., Tseng M. T., Miller F. N. (1989). A comparison of the effects of photodynamic therapy on normal and tumor blood vessels in the rat microcirculation.. Radiat Res.

[OCR_01086] Selman S. H., Kreimer-Birnbaum M., Chaudhuri K., Garbo G. M., Seaman D. A., Keck R. W., Ben-Hur E., Rosenthal I. (1986). Photodynamic treatment of transplantable bladder tumors in rodents after pretreatment with chloroaluminum tetrasulfophthalocyanine.. J Urol.

[OCR_01080] Selman S. H., Kreimer-Birnbaum M., Goldblatt P. J., Anderson T. S., Keck R. W., Britton S. L. (1985). Jejunal blood flow after exposure to light in rats injected with hematoporphyrin derivative.. Cancer Res.

[OCR_01067] Selman S. H., Kreimer-Birnbaum M., Klaunig J. E., Goldblatt P. J., Keck R. W., Britton S. L. (1984). Blood flow in transplantable bladder tumors treated with hematoporphyrin derivative and light.. Cancer Res.

[OCR_01074] Selman S. H., Milligan A. J., Kreimer-Birnbaum M., Keck R. W., Goldblatt P. J., Britton S. L. (1985). Hematoporphyrin derivative photochemotherapy of experimental bladder tumors.. J Urol.

[OCR_01063] Shulok J. R., Klaunig J. E., Selman S. H., Schafer P. J., Goldblatt P. J. (1986). Cellular effects of hematoporphyrin derivative photodynamic therapy on normal and neoplastic rat bladder cells.. Am J Pathol.

[OCR_01092] Star W. M., Marijnissen H. P., van den Berg-Blok A. E., Versteeg J. A., Franken K. A., Reinhold H. S. (1986). Destruction of rat mammary tumor and normal tissue microcirculation by hematoporphyrin derivative photoradiation observed in vivo in sandwich observation chambers.. Cancer Res.

[OCR_01098] Tseng M. T., Reed M. W., Ackermann D. M., Schuschke D. A., Wieman T. J., Miller F. N. (1988). Photodynamic therapy induced ultrastructural alterations in microvasculature of the rat cremaster muscle.. Photochem Photobiol.

[OCR_01104] Tseng M. T., Schuschke D. A., Reed M. W., Harty J. I., Wieman T. J., Fingar V. H. (1991). The influence of photodynamic therapy on the ultrastructure of the normal rat bladder.. J Photochem Photobiol B.

[OCR_01114] Wieman T. J., Mang T. S., Fingar V. H., Hill T. G., Reed M. W., Corey T. S., Nguyen V. Q., Render E. R. (1988). Effect of photodynamic therapy on blood flow in normal and tumor vessels.. Surgery.

[OCR_01120] Wolf P., Rieger E., Kerl H. (1993). Topical photodynamic therapy with endogenous porphyrins after application of 5-aminolevulinic acid. An alternative treatment modality for solar keratoses, superficial squamous cell carcinomas, and basal cell carcinomas?. J Am Acad Dermatol.

[OCR_01110] van der Veen N., van Leengoed H. L., Star W. M. (1994). In vivo fluorescence kinetics and photodynamic therapy using 5-aminolaevulinic acid-induced porphyrin: increased damage after multiple irradiations.. Br J Cancer.

